# Mapping the burden of severe forms of epidermolysis bullosa – Implications for patient management

**DOI:** 10.1016/j.jdin.2023.02.016

**Published:** 2023-03-29

**Authors:** Jemima E. Mellerio, Dimitra Kiritsi, M. Peter Marinkovich, Natividad Romero Haro, Kellie Badger, Meena Arora, Marc A. Dziasko, Mansi Vithlani, Anna E. Martinez

**Affiliations:** a St John’s Institute of Dermatology, Guy’s and St Thomas’ NHS Foundation Trust, London, UK; b Department of Dermatology, Medical Center-University of Freiburg, Faculty of Medicine, Freiburg, Germany; c Department of Dermatology, Stanford University School of Medicine, Stanford, California; d Veteran’s Affairs Medical Center, Palo Alto, California; e Department of Healthcare, DEBRA, Marbella, Spain; f Phoenix Children's Hospital, Phoenix, Arizona; g Amryt Pharma plc, London, UK; h Dolon Ltd, London, UK; i Department of Dermatology, Great Ormond Street Hospital for Children NHS Foundation Trust, London, UK

**Keywords:** blistering, clinical manifestations, disease burden mapping, epidermolysis bullosa, pathophysiology, wound healing

## Abstract

**Background:**

The pathophysiological processes underlying the phenotypic spectrum of severe forms of epidermolysis bullosa (EB) are complex and poorly understood.

**Objective:**

To use burden mapping to explore relationships between primary pathomechanisms and secondary clinical manifestations in severe forms of EB (junctional and dystrophic EB [JEB/DEB]) and highlight strengths and weaknesses in evidence regarding the contribution of different pathways.

**Methods:**

Literature searches were performed to identify evidence regarding the pathophysiological and clinical aspects of JEB/DEB. Identified publications and clinical experience were used to construct burden maps to visually communicate plausible connections and their relative importance by subtype.

**Results:**

Our findings suggest that most of the clinical consequences of JEB/DEB may result from an abnormal state and/or faulty skin remodeling driven by a vicious cycle of delayed wound healing, predominantly mediated through inflammation. The quantity and quality of evidence varies by individual manifestations and disease subtype.

**Limitations:**

The burden maps are provisional hypotheses requiring further validation and are limited by the published evidence base and subjectivity in clinical opinion.

**Conclusions:**

Delayed wound healing appears to be a key driver of the burden of JEB/DEB. Further studies are warranted to understand the role of inflammatory mediators and accelerated wound healing in patient management.

**Capsule Summary**

• This article describes possible links between the pathology of EB and its diverse clinical manifestations, highlighting the role of delayed wound healing.
• Recognizing the central role of delayed wound healing in EB suggests therapies that promote wound healing may provide substantial benefits extending beyond their impact on skin lesions.

## Introduction

Epidermolysis bullosa (EB) is a genetically heterogenous group of rare disorders that causes mechanical fragility and blistering of the skin and in some cases the epithelial lining of other organs, in response to minimal or no apparent trauma.^1^ EB affects approximately 1 in 17,000 live births, with 500,000 cases estimated worldwide.^2^ EB can be classified into 4 main types based on the level of skin cleavage within the dermal-epidermal junction.^1^ EB simplex (EBS) is generally the mildest and most common form of EB (accounting for 40% to 70% of cases), while junctional EB (JEB, 5% to 20% of cases) and dystrophic EB (DEB, 25% to 50% of cases) are less common and can affect the patient more severely.^1^^,^^3^ Kindler EB is very rare (<1% of cases), and although it can be severe, often a normal life expectancy is possible, as is the case for EBS. More than 30 different subtypes, inherited in either an autosomal dominant or recessive manner and with varying clinical presentations, are currently recognized.^1^

Patients with severe forms of EB (JEB and DEB) may have different disease expressions, ranging from a few, localized blisters to generalized skin and mucosal blistering/wounding, usually appearing around the time of birth.^4^ JEB severe (JEB-S) is the most severe subtype of EB with an early mortality less than 2 years of age. The most common reasons for infant fatality in JEB-S include failure to thrive, airway obstruction, and sepsis.^5^, ^6^, ^7^ Squamous cell carcinoma (SCC) is the most serious complication and major cause of death among adults living with severe forms of EB, notably those with severe recessive DEB (RDEB-S): By midadulthood up to 90% of patients with this subtype of EB will have died of metastatic SCC, despite aggressive surgical resection.^8^ In RDEB-S, there is also a tendency for blisters to heal with significant scarring, leading to joint contractures and pseudosyndactyly.^1^ Pain and pruritus are the main symptoms associated with JEB and DEB, and affected individuals generally have a poor health-related quality of life (HRQoL).^9^

Prior to June 2022, there were no approved therapies for EB, and the cornerstone of EB care focused on a combination of wound care, pain/itch management, and treating and trying to prevent or manage the multisystemic complications.^10^ As EB is an inherited condition, gene therapies could be the way forward in treating the underlying disease. However, considering the heterogeneity of genetic mutations, targeting disease pathways is likely to feature in the overall approach to developing treatments for people living with EB. There is a need to collate the sparse and disparate literature to build consensus among clinical experts on the nature of the disease pathways and in turn to direct effective patient management and therapeutic discovery.

One approach to achieve such a clinical initiative is to use disease burden mapping, a relatively new methodology, whereby an initial conceptual framework of the disease is constructed based on a review of the published literature and is refined through structured qualitative and semiquantitative discussions with clinical experts. Despite inherent issues of subjectivity, this approach shows promise in consolidating and corroborating heterogenous evidence to generate hypotheses on the clinical relevance of underlying disease mechanisms in rare diseases.^11^ It is similar in concept to that used to define aspects of the HRQoL burden of a disease to develop a disease-specific tool that will capture the salient impacts of a disease and changes in response to treatment. Here, we use burden mapping to generate hypotheses on the common pathomechanisms underlying clinical manifestations in JEB and DEB as well as their relative importance by subtype, for further validation.

## Methodology

Literature searches were performed in PubMed through to March 2022 to identify all consequences of the disease. A review of relevant publications was used to construct draft conceptual maps that visually linked specific complications, deformities, and symptoms with shared pathomechanisms. Structured discussions were then undertaken to obtain clinical expert feedback on the structure and components of the maps from six EB health care professionals (4 clinicians, 2 nurses) across geographies (Germany, Spain, UK, US). This included input on: pathological drivers of disease and organ systems affected; direction and nature of the relationships between primary pathomechanisms and downstream clinical manifestations; and disease impact on patient health outcomes and HRQoL. Interim maps were modified and validated based on repeated clinical input. Clinical authors were also asked to rate the frequency of pathophysiological changes and clinical manifestations in each of the most common subtypes of JEB and DEB on a 5-point scale of 0 (absent) to 4 (very high).

## Results

### Overview of cutaneous and extracutaneous involvement

**Fig 1:**
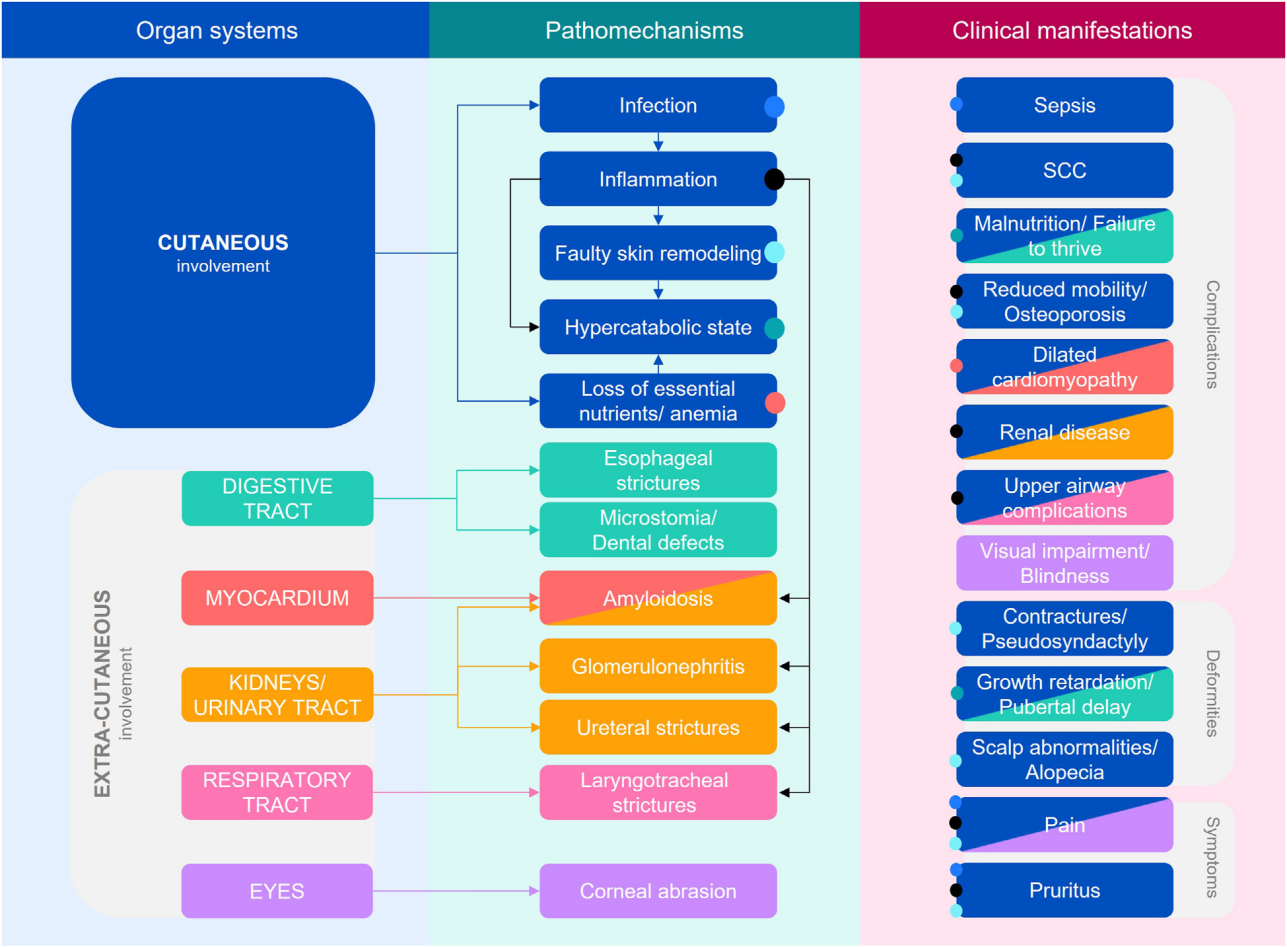
Burden map of severe forms of EB, showing that the skin is highly involved in both cutaneous and extracutaneous clinical manifestations of JEB/DEB: The figure provides a visual representation of the organ systems involved (*left panel*), the primary pathomechanisms (*middle panel*) and the secondary clinical manifestations (*right panel*) in JEB/DEB. Boxes are shaded according to the organ system(s) involved (eg, *blue* indicates cutaneous involvement) and *colored arrows* show the links between organ systems and pathomechanisms; *black arrows* indicate links between different pathomechanisms (eg, the contribution of inflammation to amyloidosis). The clinical manifestations are colored according to the main organ system(s) involved, while the *colored dots* indicate the pathomechanisms involved. For example, cutaneous involvement can lead to infection which in turn is manifest as sepsis, pain, and pruritus (*blue dots*), while the effects of inflammation (resulting from cutaneous involvement) can be manifest as SCC, reduced mobility/osteoporosis, renal disease, upper airway complications, pain, and pruritus (*black dots*). *EB*, Epidermolysis bullosa; *SCC*, squamous cell carcinoma.

A burden map of severe forms of EB identified JEB and DEB as being largely associated with cutaneous involvement and to a lesser extent the digestive, cardiovascular, nephrourological, respiratory, and visual systems (Fig 1). While effects on extracutaneous function were found to be primarily mediated directly through blistering/wounding in the epithelial lining of the organs in question, cutaneous blistering/wounding via dysregulated systemic inflammation was also implicated.

### Spotlight on cutaneous involvement

**Fig 2:**
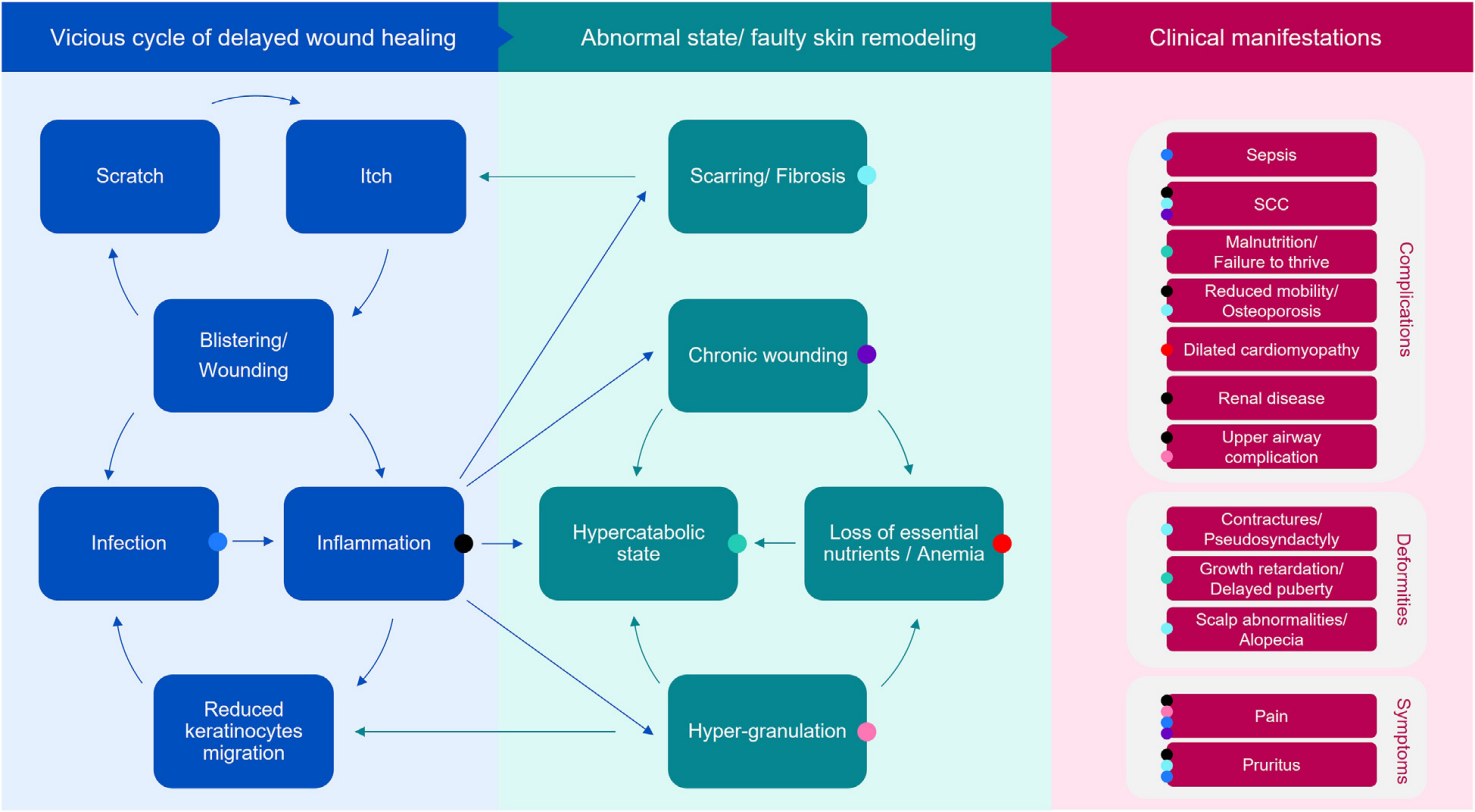
Burden map of cutaneous involvement in severe forms of EB, highlighting the underlying role of delayed wound healing in clinical manifestations of JEB/DEB. The figure provides a visual representation of the primary pathomechanisms (*left and middle panels*) and secondary clinical manifestations (*right panel*) arising from cutaneous involvement in JEB/DEB. A viscous cycle of delayed wound healing arising from disordered physiological processes are shown to evolve into an abnormal state/faulty skin remodeling – predisposing patients to serious and potentially fatal complications, deformities, and symptoms. *Arrows* indicate the links between the different pathomechanisms, while the *colored dots* indicate the pathomechanisms underlying the clinical manifestations. For example, an itch-scratch-blister cycle (applicable *blue arrows*) contributes to chronic wounding manifesting in SCC and pain (*purple dots*); chronic wounding can in turn lead to a hypercatabolic state, as a result of excess energy expenditure to regulate body temperature, and/or a loss of nutrients/anemia, further adding to a hypercatabolic state (applicable *green arrows*). *DEB*, Dystrophic EB; *EB*, epidermolysis bullosa; *JEB*, junctional EB; *SCC*, squamous cell carcinoma.

The pathomechanisms linking cutaneous blistering/wounding to clinical manifestations in JEB and DEB was then investigated in a more detailed burden map focusing on cutaneous involvement (Fig 2). Widespread unremitting blistering/wounding was reported to trigger an aberrant signaling cascade ensuing a vicious cycle of delayed wound healing. As recently reviewed by Tartaglia et al,^12^ accumulating evidence in RDEB points to disordered physiological processes arising in the final 3 stages of wound healing – inflammation, regeneration, and maturation.

**Fig 3:**
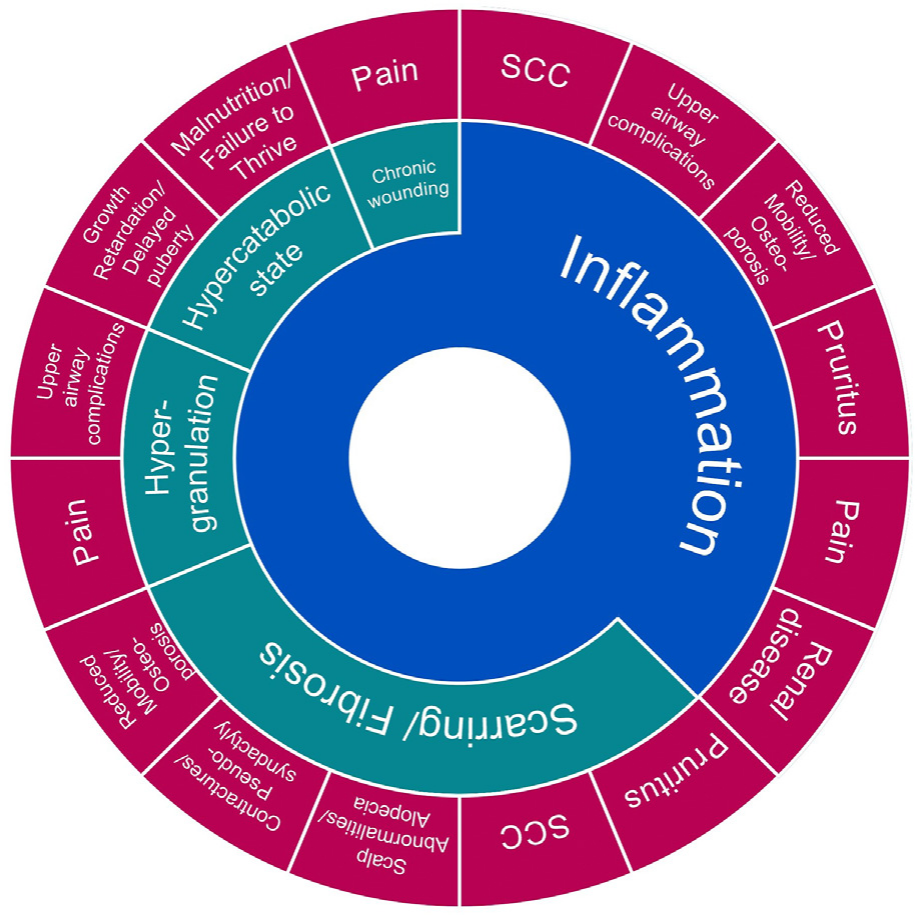
Burden map of cutaneous involvement in severe forms of EB, showing the clinical manifestations in JEB/DEB are predominantly mediated through inflammation: The figure illustrates how the clinical manifestations of JEB/DEB (*pink*) arise directly as a result of inflammation (*blue*), as is the case for SCC, upper airway complications etc. or indirectly via inflammation-driven abnormal state/faulty skin remodeling (*green*), as is the case for scalp abnormalities, contractures etc. In addition, some clinical manifestations arise both directly from inflammation and via faulty skin remodeling (eg, SCC, pain, and pruritus). *DEB*, Dystrophic EB; *EB*, epidermolysis bullosa; *JEB*, junctional EB; *SCC*, squamous cell carcinoma.

Inflammation is a constant in nonhealing wounds and is implicated, either directly or indirectly, in the majority of the clinical manifestations associated with severe forms of EB (Fig 3). Compromised epidermal-dermal adhesion and subsequent blistering/wounding as a result of the underlying genetic defects favors microbial colonization and infection that, together with repetitive injury to the skin barrier, increase and prolong the initial inflammatory response. Perturbed inflammation prevents keratinocyte migration/differentiation and regeneration of the epithelial barrier, leading to wound recurrence. At the same time, itch occurring in blistered or wounded sites sets off an itch-scratch-blister cycle, further exacerbating the risk of infection and unresolved inflammation.^13^ A persistent state of inflammation slows down the maturation of wounds, resulting in chronic wound and scar formation/fibrosis or hypergranulation. In addition, presence of open wounds results in excess energy expenditure to maintain normal body temperature and direct loss of body fluids/essential nutrients, resulting in a hypercatabolic state. Abnormal state/faulty remodeling in turn favors the development of downstream complications, deformities, and symptoms characteristic of this group of debilitating and life-threatening disorders.

The implications of these clinical manifestations were highlighted in the identified published literature and corroborated by the clinical experts. Wound burden and care were reported to have a significant impact on daily activities and HRQoL of patients. Painful blistering/wounding on the soles of the feet and/or complications of significant hyperkeratosis/scarring/fibrosis can impact an individual’s ability to walk or function^14^; as a result, many patients living with severe subtypes of JEB and DEB are registered disabled. Bathing and dressing changes can take hours each day and are often painful, anxiety provoking experiences for patients.^15^ Furthermore, pain, itch, and discomfort overshadow much of a patient’s life and they may continually experience stress as they seek to avoid the everyday physical contact that will damage their skin.^15^ Most children with severe subtypes of JEB and DEB are unable to experience a normal childhood. Furthermore, patients are frequently hospitalized and may miss blocks of education or work as a result of their condition.^15^ Families and carers of patients are also affected, as their lives can feel dominated by the routine of wound care and specialist visits.

### Contribution of pathways in the burden of severe forms of EB by subtype

**Fig 4:**
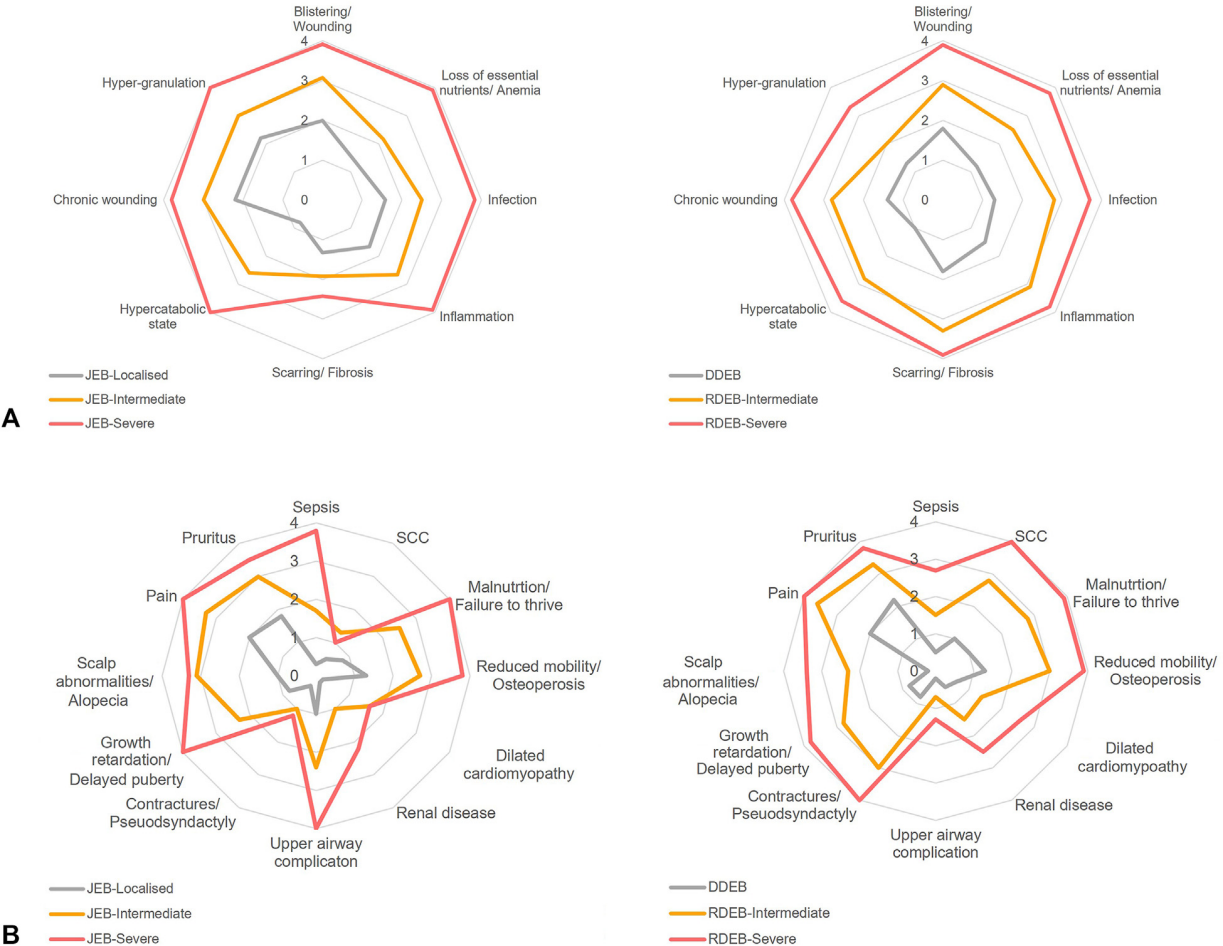
Frequency of pathophysiological changes and clinical manifestations for JEB and DEB, according to disease severity. These figures show the clinical authors’ ratings of the frequency of (**A**) pathophysiological changes and (**B**) clinical manifestations in patients with JEB (*left panel*) and DEB (*right panel*), as rated on a 5-point scale: 0 = Absent; 1 = Low; 2 = Medium; 3 = High; and 4 = Very high. Comparisons across severity subtypes for both JEB and DEB indicate an increasing frequency of all pathophysiological changes with disease severity. A similar pattern was seen for most clinical manifestations with the exception of SCC, dilated cardiomyopathy, and contractures/pseudosyndactyly, which were given a low rating for JEB-S, reflecting the fact that most patients die in infancy before the development of such manifestations. Note: clinicians did not rate the frequency of reduced keratinocyte migration as this is a laboratory measure that is not assessed in routine clinical practice. *DDEB*, Dominant dystrophic epidermolysis bullosa; *DEB*, dystrophic epidermolysis bullosa; *JEB*, junctional epidermolysis bullosa; *RDEB*, recessive dystrophic epidermolysis bullosa; *SCC*, squamous cell carcinoma.

Clinical expert assessments of the main pathomechanisms (Fig 4, *A*) and clinical manifestations (Fig 4, *B*) found that overall, these occurred more frequently in severe subtypes, with some notable differences within JEB and between JEB and DEB. JEB-S was less frequently associated with pubertal delay and SCC compared with intermediate JEB, despite a higher reported incidence of underlying pathomechanisms. This finding reflects the fact that most patients with JEB-S die in infancy, meaning there is insufficient time to develop these clinical features. Hypergranulation was most frequently reported for JEB-S. Granulation tissue bleeds easily and profusely making infants more susceptible to anemia, serious infections, and loss off body fluids/essential nutrients. Additionally, a build-up of granulation tissue in airways can cause difficulty in breathing. The highly fibrotic environment reported in RDEB versus JEB is consistent with higher ratings for downstream SCC and joint contractures/pseudosyndactyly in RDEB, in particular RDEB-S.

**Fig 5:**
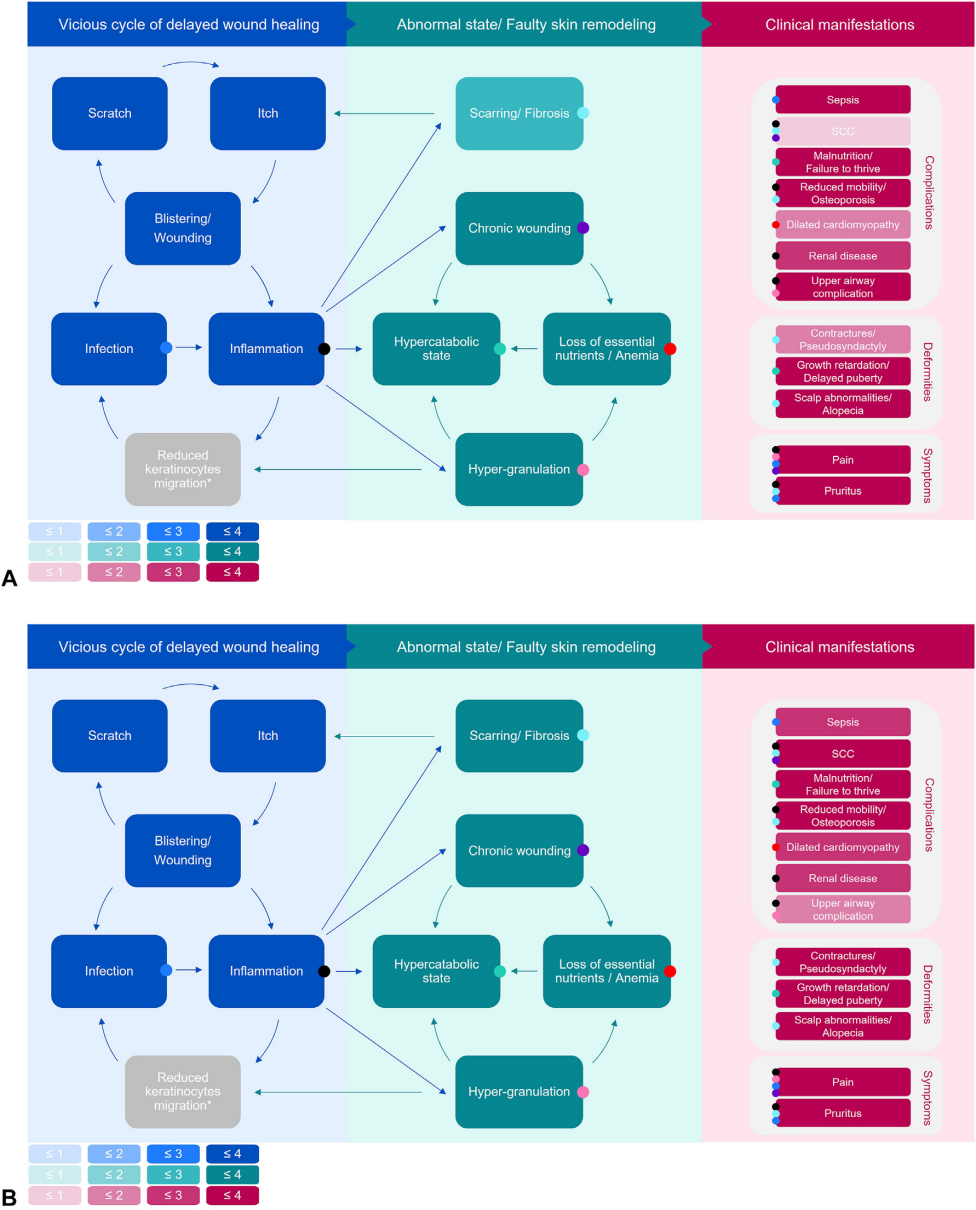
Dedicated burden maps of cutaneous involvement for JEB-S and DEB-S showing the contribution of different pathways in patient outcomes by subtype. The figure provides a visual representation of the frequency of primary pathomechanisms (*left and middle panels*) and the frequency of secondary clinical manifestations (*right panels*) arising from cutaneous involvement in (**A**) JEB-S and (**B**) DEB-S through proportionately shaded boxes. Boxes were shaded based on clinical authors’ ratings from Fig 4 as shown in the key. For example, in JEB-S, the frequency of Escarring/fibrosis is shown to be medium-to-high [] resulting in an absent-to-low frequency of SCC [] compared to high-to-very high frequency of both scarring/fibrosis [] and SCC [] in DEB-S. *DEB*, Dystrophic epidermolysis bullosa; ∗Note: clinicians did not rate the frequency of reduced keratinocyte migration as this is a laboratory measure that is not assessed in routine clinical practice. *DEB-S*, severe dystrophic epidermolysis bullosa; *JEB-S*, severe junctional epidermolysis bullosa; *SCC*, squamous cell carcinoma.

Clinical expert ratings of the frequency of the main pathophysiological changes and clinical manifestations can be superimposed onto the burden map of cutaneous involvement to produce dedicated burden maps for each subtype. Fig 5 graphically represents the contribution of different pathways in patient outcomes in JEB-S (Fig 5, *A*) and DEB-S (Fig 5, *B*) through proportionately shaded boxes.

## Discussion

The pathophysiological processes underlying the phenotypic spectrum of severe forms of EB are complex and poorly understood. In this study, we used burden mapping as a means of gaining valuable insight into possible connections between primary pathomechanisms and secondary clinical manifestations (complications, deformities, and symptoms). Our findings propose a role for delayed wound healing in the burden of severe forms of EB – predominantly mediated through inflammation. Much of the evidence is available in the published literature but this tends to focus on individual clinical features or a given EB subtype, rather than considering the common pathomechanisms underlying the range of clinical manifestations and/or the relative contribution of different pathways by subtype. We suggest burden mapping as a useful tool in understanding and building consensus among clinical experts on the etiology and severity of different diseases. This is particularly important in the context of often poorly understood and under-researched rare diseases, where defined and validated outcome measures are frequently lacking. Having generated plausible links through this approach based on published literature and clinical experience, further research efforts can be directed to yield evidence where this may be limited or where uncertainties remain. This in turn should help in the design of clinical trials and the advent of new therapies.

In the absence of approved EB-specific therapies, patient care has centered on protection from minor trauma and friction, bandaging, and prevention of infection.^11^ However, given that delayed wound healing appears to be a key mediator in the burden of JEB and DEB, as observed in the burden mapping results reported here, approaches that modulate inflammation and accelerate wound healing could be clinically meaningful and potentially benefit patients and their caregivers. For example, results of a recent phase 3 trial in patients with severe forms of EB found that the topical gel Oleogel-S10 (birch bark extract, also referred to as birch triterpenes) demonstrated accelerated wound healing by achieving complete target wound closure in 41.3% of target wounds compared to 28.9% with vehicle control gel (*P* = .013) within 45 days.^16^ This accelerated wound healing with Oleogel-S10 resulted in a reduction in total body wound burden.^16^^,^^17^ On the basis of these results, Oleogel-S10 (birch bark extract) has now been approved for treatment of partial thickness wounds associated with JEB and DEB in the EU and UK. Another recent publication reporting the phase 3 trial evaluating the redosable topical gene therapy beremagene geperpavec, in patients with DEB reported complete wound healing occurred in 67% of the wounds exposed to beremagene geperpavec as compared with 22% of those exposed to placebo (*P* = .002).^18^

A number of further therapeutic interventions are being explored.^4^^,^^19^ These include gene replacement therapies, gene correction therapies, RNA-based therapies, and other therapies focusing on halting disease progression by targeting inflammation and fibrosis. Gene replacement therapies aim to replace defective genetic material in the skin^20^ and have shown particular promise in patients with JEB carrying mutations in *LAMB3*.^20^, ^21^, ^22^ Gene correction using site-directed technologies such as CRISPR/Cas9 have been used to restore *KRT5* in EBS^23^ and *COL7A1* in dominant DEB.^24^ RNA-based therapies are also in development using established principles of antisense oligonucleotides and RNA trans-splicing.^25^ Other gene- and protein-directed approaches are also under investigation,^23^, ^25^, ^26^ and the clinical community awaits the outcomes of this research with anticipation.

Our study has several limitations, which should be considered when interpreting the findings. First, evidence for the clinical manifestations of JEB and DEB that we consider largely reflects published literature and hence may not include those that have potentially not been described. From our clinical experience of managing these patients we believe that most of the major drivers of disease burden have been reported, but given the interpatient diversity, it is possible that some features seen in a minority of patients have not been captured. Second, our study relies on the subjective assessment of the 6 clinical authors involved in the study. Involvement of a larger panel of clinicians may have helped ensure that the findings are relevant for all patients with JEB and DEB. However, the panel included clinicians from specialist centers in the US, Germany, Spain, and the UK and therefore is broadly representative of the clinical experience in Europe and the US. Finally, our findings are provisional hypotheses; further studies are needed to validate the role of delayed wound healing in the burden of JEB and DEB.

## Conclusions

In the present study, we used clinical expert opinion along with published evidence to link pathomechanisms underlying clinical manifestations in severe forms of EB. Our resultant burden maps suggest delayed would healing as a key driver of the burden of JEB and DEB, underpinned by dysregulated inflammation.

## Conflicts of interest

Jemima E. Mellerio has undertaken paid consultancy and speaker for Amryt Pharmaceuticals DAC and Krystal Biotech Inc. Dimitra Kiritsi has undertaken paid consultancy for Amryt Pharmaceuticals DAC, Rheacell GmbH and FibRx Derm Inc; is a founder at Crowd Pharma GmbH. M. Peter Marinkovich has undertaken paid consultancy for Amryt Pharmaceuticals DAC; and is an investigator for Krystal Biotech Inc, Abeona Therapeutics Inc and Castle Creek Biosciences, through funding administered by Stanford University Office of Sponsored Research. Natividad Romero Haro has undertaken paid consultancy for Amryt Pharmaceuticals DAC, Kellie Badger has undertaken paid consultancy for Amryt Pharmaceuticals DAC, Meena Arora is an employee at Amryt Pharmaceuticals DAC. Marc A. Dziasko has undertaken paid consultancy services to Amryt Pharmaceuticals DAC. Mansi Vithlani has undertaken paid consultancy services to Amryt Pharmaceuticals DAC. Anna E. Martinez has undertaken paid consultancy for Amryt Pharmaceuticals DAC.
